# Early life exposures and risk of adult respiratory disease during 50 years of follow-up

**DOI:** 10.1007/s10654-020-00626-3

**Published:** 2020-04-08

**Authors:** Carl J. Johansson, Peter M. Nilsson, Claes Ignell

**Affiliations:** 1 Department of Internal Medicine, Danderyd Hospital, Stockholm, Sweden; 2 Department of Clinical Sciences, Internal Medicine, Skane University Hospital, Lund University, Malmö, Sweden; 3 Department of Obstetrics and Gynaecology, Helsingborg Hospital, Helsingborg, Sweden

**Keywords:** Epidemiology, Lung, Pregnancy, Respiratory disease, Sedatives, Smoking

## Abstract

Adult health is influenced by factors during fetal life affecting organ development and birth weight. We aimed to study such factors in relation to adult respiratory disease (ARD) risk. The Helsingborg Birth Cohort, Sweden, contributed baseline data collected by medical staff through clinical examination and questionnaires on maternal and birth characteristics 1964–1967. Register linkages were performed with completions of data on ARD by ICD 8–10 classifications (1969–2016), and/or ARD-related drug usage (2005–2016) enabling a 50-year follow-up time. Cox proportional hazard regression analyses were made to adjust for potential confounders, adjusted hazard ratio (aHR). A total of 3675 mothers and their offspring were included. Female offspring showed higher frequency of ARD than males, aHR 1.5 (95% CI 1.3–1.8). Maternal use of sedatives during second trimester, aHR 2.2 (95% CI 1.4–3.4), and maternal smoking during most of pregnancy, aHR 1.2 (95% CI 1.0–1.4), were associated with offspring ARD. Stratified by sex, large-for-gestational-age, aHR 1.4 (95% CI 1.0–1.9), was significantly associated with ARD in female offspring along with maternal sedative use during second trimester and maternal smoking during most of pregnancy. Maternal sedative use during second trimester or all trimesters were the only significant risk factors for male offspring. In conclusion, maternal sedative use in second trimester was independently associated with subsequent respiratory disease in adult offspring irrespective of sex.

**Electronic supplementary material:**

The online version of this article (10.1007/s10654-020-00626-3) contains supplementary material, which is available to authorized users.

## Introduction

Asthma and chronic obstructive pulmonary diseases (COPD) are among the leading causes of global mortality and morbidity, representing a heavy economic burden [1, 2]. Although largely considered smoking-related diseases, increasing evidence supports that adult respiratory health is at least partly affected by prenatal growth patterns and the intrauterine environment.

The fetal origins hypothesis, according to Barker et al. [3, 4], suggested that low birth weight for gestational age is associated with adult diseases such as ischemic heart disease and high blood pressure [5] due to the mal-development of organ anatomy and function predisposing for disease. Several studies have shown similar associations between low birth weight and adult COPD [6–9], impaired lung function [10–12], and asthma [9, 12, 13]. Less studies have, however, reported the effects of preterm birth or intrauterine growth retardation (IUGR) on the development of adult respiratory disease (ARD) in general [14].

According to the developmental plasticity hypothesis presented by Gluckman and Hanson the developing fetus can be subjected to both adaptive and non-adaptive/disruptive responses from changes in the intrauterine environment [15]. Adaptive responses include, for example, reduced fetal growth or low birth weight in adaption to a predicted scarce postnatal environment, which implied an evolutionary benefit in the case of a match with predicted postnatal environment, but in the case of a mismatch an increased risk of disease. Importantly these predictive adaptive responses occur not only in low birth weight children, but across the entire birth weight range. In the case of disruptive responses, these respond to a toxic or severe insult interfering with the embryonic development causing dysfunction of the fetus and new-born child. Examples are teratogenic drugs, maternal cigarette smoking, hypoxia, and pre-eclampsia causing IUGR, but also maternal obesity or over-nutrition resulting in large-for-gestational-age (LGA) births (macrosomia) [15].

IUGR is currently diagnosed by ultrasound and thus hard to reproduce in historical cohorts. By adjusting birth weight for gestational age it is however possible to investigate long-term health consequences of infants born small-for-gestational-age (SGA).

The *aim* of this explorative, cohort follow-up study of more than 4000 children was to expand the knowledge of how alterations in the intrauterine environment, also linked to growth retardation, affect risk of ARD.

Our working hypothesis is that maternal smoking and subsequent IUGR/SGA along with other early life insults constitutes risk factors for ARD. We therefore examined if an association exists between gestational risk factors/markers, such as maternal smoking and drug treatment, as well as prematurity and SGA of the child, on the one hand, with early ARD before age of 50 years on the other hand.

## Subjects and methods

### Subjects

We used data from a prospective study based on the Helsingborg Birth Cohort (HbgBC). The original HbgBC data collection was based on 4982 diagnosed pregnancies (missing, *n *= 5) of which 4091 singleton pregnancies were included in the cohort delivered from February 1st 1964 to January 31th 1967 at the Department of Obstetrics, Helsingborg Hospital, Sweden [16]. Data was collected by structured questionnaires completed by practicing gynaecologists, patients, midwifes, obstetricians and paediatricians clinically examining all infants [17].

**Fig. 1:**
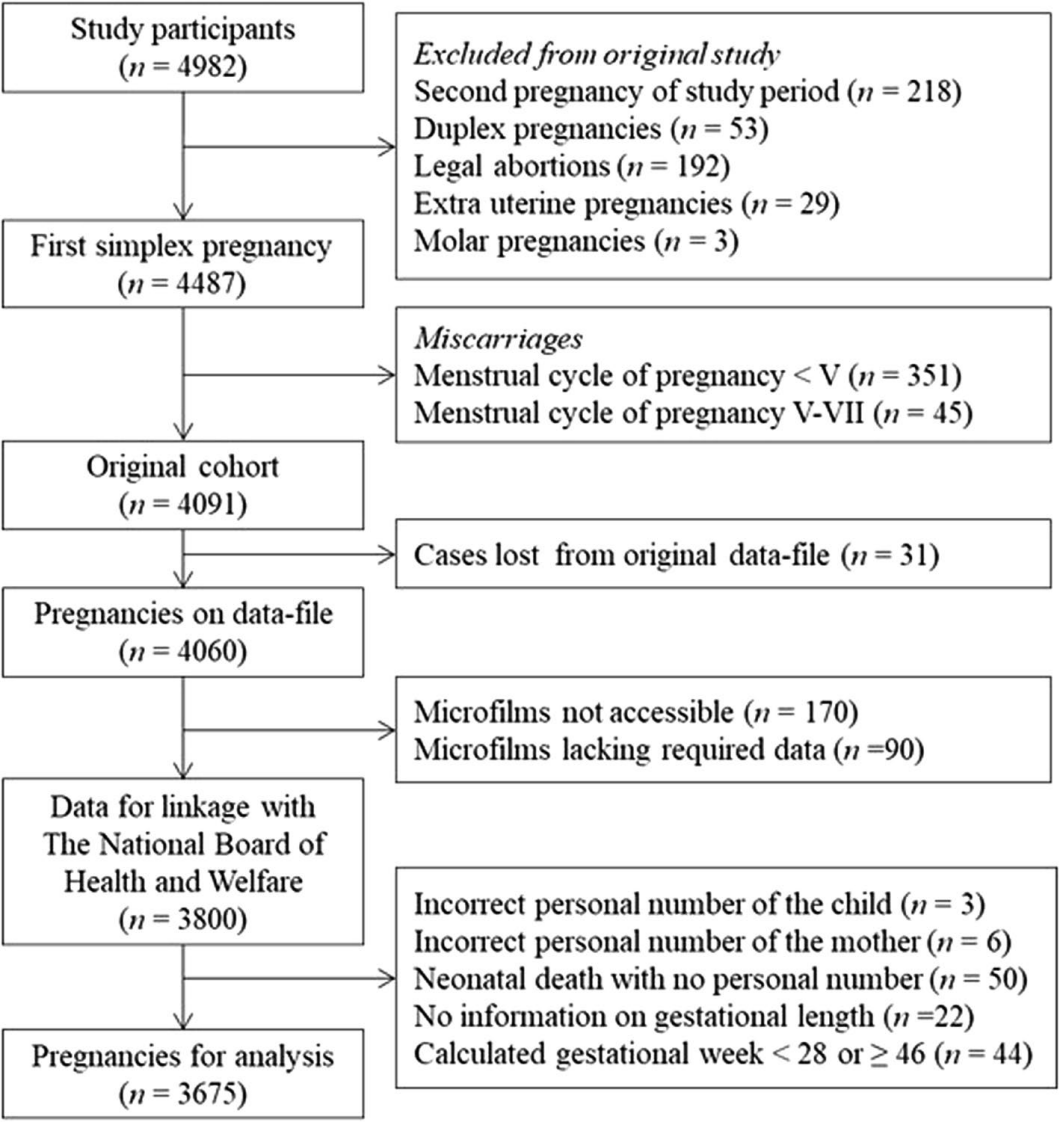
Flowchart of the study population

Thirty-one women were lost in later data analysis (1995) for technical reasons, and 170 women lacked medical records in the archive enabling data from 3890 women and children to be used in this study. Women with medical records lacking data regarding gestational week of delivery or lacking information regarding smoking history were categorized as missing (*n *= 90). At linkage with national registers, nine mothers or children were found to have incorrect personal numbers. A total of 116 pregnancies were excluded after register linkage; involving women that gave birth to offspring which deceased intra-partum or during the neonatal period, or women delivering before gestational week 28 + 0 or later than gestational week 45 + 6 or lacked information on gestational week of delivery. Thus, the final study sample included 3675 singleton births (Fig. 1). Gestational week of delivery was calculated from self-reported date of last menstrual period obtained from the maternal healthcare records. Owing to the inaccessibility of intra-uterine growth curves from the period 1964 to 1967, expected birth weight by gestational length and sex was calculated using a modern algorithm elaborated by Marsal et al. [18]. Deviation of actual birth weight from expected birth weight was calculated (%) and the children from the 10th percentile of lowest birth weight deviation within this cohort was defined as SGA.

### Register follow-up

A record linkage analysis was carried out linking the local pregnancy register (HbgBC 1964–1967) with data on hospital-derived diagnoses by use of the National Patient Register (since 1969) and the Cause of Death Register (since 1969) obtained from the National Board on Health and Welfare, Stockholm, until December 31st 2016. Hospital-derived diagnoses (for inpatients, and hospital policlinic patients) were used according to the International Classification of Diseases (ICD 8–10) and were linked for *chronic bronchitis* (ICD-10: J42.9; ICD-9: 4918, 4919; ICD-8: 466, 491), *emphysema* (ICD-10: J43.0–J43.9; ICD-8,9: 492), *acute and chronic asthma* (ICD-10: J45.0–J.45.9, J46.9; ICD-9: 4930, 4931, 4939; ICD-8: 490, 493), *COPD* (ICD-10: J44.0–J44.9; ICD-9: 4912, 4918, 496; ICD-8: 5192, 5196, 491, 466) and *bronchiectasies* (ICD-10: J47.9; ICD-9: 494; ICD-8: 518).

Since there is no available national diagnostic register from primary care, drug usage according to the Anatomical Therapeutic Chemical Classification System (ATC) were obtained from the Swedish Drug Register (since 2005) for ARD, such as adrenergic inhalations (R03A), other inhalations including steroids (R03B), adrenergic systemic drugs (R03C), and other systemic drugs (R03D), considered as surrogate markers of respiratory disease.

### Statistical methods

All statistical analyses were performed using IBM SPSS Statistics (version 25.0, IBM Corp, Armonk, NY, USA). Data are displayed as *n* (%) for categorical variables and as mean and standard deviation (SD) for continuous variables. Chi-2 analysis and Fischer’s exact test were used to compare group frequencies of categorical variables. Student’s t test, Mann–Whitney U-test and one-way ANOVA were used to compare means of continuous variables. Level of significance was set to *P *< 0.05, but *P *< 0.1 was considered as trend for inclusion in multivariable regression. If group comparisons for tested variables between ARD cases and reference group (non-affected) were significant or showed a trend, and if sample size was sufficient, Cox proportional hazard regression was used to calculate unadjusted hazard ratio (HR) and 95% confidence interval (95% CI). Confounders were selected by stepwise forward regression and common cause approach for adjusted analysis to determine adjusted hazard ratio (aHR) and 95% CI’s for ARD. Tolerance was calculated using linear regression to examined potential collinearity between independent variables.

ARD cases were defined as offspring that either received a diagnosis of respiratory disease (acute bronchitis not included) or prescriptions of medical drugs used to treat these conditions. Variables tested for associations were: maternal age (year), maternal weight (kg), gestational week of delivery (weeks), born during winter season (1st November–31st January: yes/no), prematurity (yes/no), small for gestational age (SGA)/appropriate for gestational age (AGA)/large for gestational age (LGA), working/studying during pregnancy (yes/no), birth height (cm), maternal use of sedative drug medication during pregnancy (none/yes, during part of pregnancy/yes, all pregnancy), primiparity (yes/no), placental weight (grams), smoking in pregnancy (none/during first trimester/throughout entire pregnancy), maternal infection during pregnancy (none/yes, any of following: Upper Respiratory tract Infection (URI) with fever ≥ 39 °C; Urinary Tract Infection (UTI); URI + UTI; or other infection with fever), maternal antibiotic treatment during pregnancy (yes/no), maternal other antimicrobial treatment during pregnancy (yes/no), and maternal mode of delivery (vaginal; vacuum extraction; or caesarean section).

## Results

Altogether, 749 (20.4%) offspring individuals either received a diagnosis of ARD or a prescription of drugs used to treat such conditions. Among these individuals, 181 (4.9%) received a hospital-derived diagnosis only and 137 (3.7%) received both a hospital-derived diagnosis and drug prescription (since 2005), the rest only a drug prescription. The mean year of first recorded ARD diagnosis as available from the registries was 2000 (median year 2005) and mean age 33.6 (median 39). Unfortunately no detailed information for onset of disease was available. The most common prescribed drug was beta-receptor agonist inhalators, and the most common diagnosis was asthma (acute and/or chronic), see Supplemental Figures S1 and S2.

### Characteristics of study participants

Characteristics of the study population are shown in Table 1. Out of 3675 pregnancies, 1786 female and 1889 male children were born. Offspring affected by ARD were more commonly exposed to maternal use of sedative drugs during pregnancy while pre-eclampsia, by the time of labour, was less common. Significantly more female offspring were diagnosed with ARD or prescribed drug treatment than male offspring, 24.6% versus 16.4% (*P *< 0.001).

**Table 1:** Maternal and offspring characteristics of the study population from the Helsingborg Birth Cohort (HbgBC), Sweden, with or without adult respiratory disease

	No, *n *= 2926		Yes, *n *= 749		*P*^a^
	Mean (SD)	Missing	Mean (SD)	Missing	
Maternal age (years)	26.4 (5.7)	0	26.2 (5.7)	0	0.2
Maternal weight (kg)	57.7 (8.5)	377	57.8 (9.0)	83	0.7
Placental weight (g)	609.1 (118.8)	129	606.7 (123.9) (123.9)	34	0.6
Maternal smoking, *n* (%)		1		0	**0.04**
Non-smoker	1524 (52.1)		362 (48.3)		
Smoking during 1st trimester	128 (4.4)		25 (3.3)		
Smoking during most of pregnancy	1273 (43.5)		362 (48.3)		
Maternal infections, *n* (%)		0		0	0.4
No infections during pregnancy	2348 (80.2)		611 (81.6)		
Infections during pregnancy infection (URI), > 39 °C	578 (19.8)		138 (18.4)		
Maternal use of penicillin, *n* (%)		2		0	0.4
No penicillin used during pregnancy	2577 (88.1)		667 (89.1)		
During 1st trimester	64 (2.2)		20 (2.7)		
During 2nd trimester	68 (2.3)		21 (2.8)		
During 3rd trimester	168 (5.7)		34 (4.5)		
During all trimesters	4 (0.1)		0 (0.0)		
Maternal use of other antibiotics, *n* (%)		3		0	0.6
No other antibiotics used during pregnancy	2533 (86.7)		659 (88.0)		
During 1st trimester	56 (1.9)		13 (1.7)		
During 2nd trimester	74 (2.5)		21 (2.8)		
During 3rd trimester	198 (6.8)		44 (5.9)		
During all trimesters	9 (0.3)		0 (0.0)		
Maternal use of sedatives, *n* (%)		172		44	**0.009**
No sedatives used during pregnancy	1521 (52.2)		366 (51.9)		
During 1st trimester	95 (3.4)		18 (2.6)		
During 2nd trimester	37 (1.3)		21 (3.0)		
During 3rd trimester	998 (36.2)		266 (37.7)		
During all trimesters	103 (3.7)		34 (4.8)		
Nulliparous, *n* (%)	1349 (46.1)	0	363 (48.5)	0	0.3
Birth weight categories, *n* (%)		1		0	0.3
Small for gestational age (SGA)	284 (9.7)		84 (11.2)		
Appropriate for gestational age (AGA)	2355 (80.5)		584 (78.0)		
Large for gestational age (LGA)	286 (9.8)		81 (10.8)		
Prematurity (< 37 weeks), *n* (%)	146 (5.0)	0	43 (5.7)	0	0.4
Preeclampsia by time of labour, *n* (%)	124 (4.4)	106	16 (2.2)	31	**0.007**
Sex, *n* (%)		0		0	**< 0.001**
Women	1346 (46.0)		440 (58.7)		
Men	1580 (54.0)		309 (41.3)		
Born during winter (1st Nov–31st Jan), *n* (%)	661 (22.6)	0	177 (23.6)	0	0.6

Means (SD) and proportions (%). *P* < 0.05 was considered significant

^a^Case group versus reference group (non-affected)

*P* < 0.05 was considered significant

Variables that were associated with elevated risk of ARD in unadjusted analysis were: maternal smoking during most of pregnancy (*P *= 0.02), maternal use of sedative drugs during second trimester (*P *= 0.001), offspring female sex (*P *< 0.001). Increasing offspring birth length was found to be protective for offspring ARD (*P *= 0.01).

Non-response analysis regarding cohort loss to follow-up showed that the mothers in the excluded group (*n *= 1100) were significantly younger (*P *= 0.004), smoked more (*P *= 0.002), suffered from infections more frequently (*P *= 0.03) and gave birth to children more often born < 2500 g *(P *= 0.01), SGA or LGA (*P *< 0.001) and more often treated for lung complications during the neonatal period (*P *≤ 0.001).

### Multivariate adjusted analyses

Multivariate adjusted analyses with offspring ARD as dependent variable and early life factors as independent variables are shown in Tables 2 (total sample) and 3 (stratified by sex). It was found that maternal smoking during most of pregnancy and female sex was significantly associated with offspring ARD. Sedative drug usage during second trimester also remained a significant risk marker for offspring ARD, irrespective of sex, and after full adjustment, aHR 2.2 (95% CI 1.4–3.4; *P *< 0.001).

**Table 2:** Univariate and adjusted associations between early life risk factors and offspring adult respiratory disease (ARD)

	HR^a^ (95% CI)	*P*	aHR^b^ (95% CI)	*P*	aHR^c^ (95% CI)	*P*
Gestational age (weeks)	1.0 (0.99–1.0)	0.9	0.99 (0.95–1.0)	0.6	0.99 (0.95–1.0)	0.6
Birth weight (100 g)	1.0 (0.99–1.0)	0.1	0.99 (0.98–1.0)	0.4	1.0 (0.98–1.0)	0.5
Birth height (cm)	1.0 (0.99–1.0)	**0.01**	1.0 (1.0–1.0)	0.2	1.0 (1.0–1.0)	0.2
Sex						
Female	1.6 (1.3–1.8)	**< 0.001**	1.5 (1.3–1.8)	**< 0.001**	1.5 (1.3–1.8)	**< 0.001**
Male	Reference		Reference		Reference	
Weight groups						
Small for gestational age (SGA)	1.2 (0.9–1.6)	0.2	1.1 (0.9–1.4)	0.3	1.1 (0.9–1.4)	0.4
Appropriate for gestational age (AGA)	Reference		Reference		Reference	
Large for gestational age (LGA)	1.1 (0.9–1.4)	0.3	1.2 (0.9–1.5)	0.2	1.2 (0.9–1.5)	0.2
Born during winter (1st Nov - 31st Jan)	1.0 (0.9–1.2)	0.8	1.0 (0.9–1.2)	0.8	1.0 (0.9–1.2)	0.8
Prematurity (< 37 weeks)	1.1 (0.8–1.5)	0.4	1.2 (0.9–1.6)	0.3	1.2 (0.9–1.6)	0.3
Maternal use of sedative drugs						
No sedatives used during pregnancy	Reference		Reference		Reference	
During 1st trimester	0.8 (0.5–1.3)	0.3	0.8 (0.5–1.3)	0.3	0.8 (0.5–1.3)	0.3
During 2nd trimester	2.2 (1.3–3.3)	**0.001**	2.2 (1.4–3.4)	**0.001**	2.2 (1.4–3.4)	**< 0.001**
During 3rd trimester	1.1 (0.9–1.3)	0.2	1.1 (1.0–1.3)	0.2	1.1 (0.9–1.3)	0.3
During all trimesters	1.3 (0.9–1.9)	0.1	1.4 (1.0–2.0)	0.1	1.4 (1.0–2.0)	0.08
Maternal infections						
No infections during pregnancy	Reference		Reference		Reference	
Any infection during pregnancy	0.9 (0.8–1.1)	0.6	0.9 (0.8–1.1)	0.5	0.9 (0.8–1.1)	0.6
Maternal smoking						
No	Reference		Reference		Reference	
Smoking during 1st trimester	0.9 (0.6–1.3)	0.5	0.8 (0.5–1.3)	0.4	0.8 (0.5–1.3)	0.4
Smoking during most of pregnancy	1.2 (1.0–1.4)	**0.02**	1.2 (1.0–1.4)	**0.01**	1.2 (1.0–1.4)	**0.02**
Mode of delivery						
Vaginal	Reference		Reference		Reference	
Sectio	1.1 (0.7–1.9)	0.7	1.3 (0.8–2.2)	0.3	1.3 (0.8–2.2)	0.3
Vacum extraction	1.1 (0.7–1.6)	0.8	1.0 (0.7–1.6)	0.9	1.0 (0.7–1.6)	0.9
Other	1.0 (0.5–2.3)	0.9	0.9 (0.4–2.2)	0.9	0.9 (0.4–2.2)	0.8

*HR* hazard ratio, *95% CI* 95% confidence interval, *aHR* adjusted hazard ratio

^a^Univariate logistic regression

^b^Model 1 (stepwise forward regression): adjusted HR for: sex, maternal sedative use, and maternal smoking

^c^Model 2 (common cause approach): adjusted HR for sex, maternal sedative use, maternal smoking, maternal age, and nulliparity

* P* < 0.05 was considered significant

**Table 3:** Multiple adjusted analysis for risk of ARD, stratified by offspring sex

	Women		Men	
	aHR^a^ (95% CI)	*P*	aHR^b^ (95% CI)	*P*
Birth height (cm)	1.0 (0.99–1.0)	0.3	1.0 (0.99–1.0)	0.5
Maternal use of sedatives				
No sedatives used during pregnancy	Reference		Reference	
During 1st trimester	0.8 (0.4–1.5)	0.5	0.7 (0.3–1.6)	0.4
During 2nd trimester	2.3 (1.3–4.1)	**0.01**	2.1 (1.1–4.2)	**0.03**
During 3rd trimester	1.1 (0.9–1.4)	0.4	1.1 (0.9–1.4)	0.4
During all trimesters	0.9 (0.5–1.6)	0.7	1.9 (1.2–2.9)	**0.01**
Maternal infections				
No infections during pregnancy	Reference		Reference	
Any infection during pregnancy	0.9 (0.7–1.2)	0.4	1.0 (0.7–1.3)	0.9
Maternal smoking				
No	Reference		Reference	
Smoking during 1st trimester	0.7 (0.4–1.3)	0.4	0.9 (0.5–1.7)	0.8
Smoking during most of pregnancy	1.3 (1.1–1.6)	**0.01**	1.1 (0.9–1.4)	0.4
Mode of delivery				
Vaginal	Reference		Reference	
Vacuum extraction	0.9 (0.5–1.7)	0.7	1.2 (0.7–2.2)	0.5
Caesarean section	1.8 (1.0–3.4)	0.05	0.7 (0.3–1.9)	0.5
Other	0.8 (0.3–2.4)	0.7	1.3 (0.3–5.2)	0.7
Birth weight categories				
Small for gestational age (SGA)	1.3 (0.9–1.7)	0.2	1.0 (0.7–1.5)	0.9
Appropriate for gestational age (AGA)	Reference			
Large for gestational age (LGA)	1.4 (1.0–2.0)	**0.03**	0.9 (0.6–1.4)	0.7

*95% CI* 95% confidence interval, *aHR* adjusted hazard ratio

^a^Adjusted for maternal age, nulliparity, maternal smoking and maternal sedative use

^b^Adjusted for maternal age, nulliparity, maternal smoking and maternal sedative use

*P* < 0.05 was considered significant

Linear regression was used to see if the data met the assumption of collinearity and indicated that multicollinearity was not a concern (The tolerance values were > 0.9 for all variables).

When stratified for offspring sex, birth length was no longer a significant risk marker, reflecting a lower risk in male offspring. However, the LGA phenotype was found to be a significant risk marker for ARD among female offspring in the adjusted analysis. Maternal sedative use during second trimester and maternal smoking during most of pregnancy were significantly associated with risk of ARD in female offspring. For male offspring only maternal sedative use during second trimester and during all trimesters were significant in the adjusted analysis.

Sub analyses of offspring diagnosed with asthma (*n *= 140) concluded maternal sedative use during second trimester and female sex as risk factors (Supplemental Table S2).

## Discussion

This observational, long-term follow-up cohort study with detailed exposure data indicates that early life factors (maternal and fetal) may influence offspring adult risk of respiratory disease. The main novel finding is that maternal use of sedative drugs during pregnancy, especially during the second trimester, is independently associated with increased risk of offspring ARD. Female sex and maternal smoking during most of pregnancy were also associated with higher risk,

To the best of our knowledge this is the first study to report an association of maternal use of sedative drugs (i.e. barbiturates or benzodiazepines) during pregnancy and offspring ARD. Previous studies have shown increased risk of respiratory events, exacerbations and hospitalization in adult COPD and asthma patients using benzodiazepines. This may partly be caused by respiratory suppression by depressing the central respiratory drive, decreasing respiratory muscle strength, increasing sleep-disordered breathing such as sleep-apnea and increasing upper airway resistance [19–21].

Although we lack data on which specific sedatives were used in this cohort there were, in fact, only limited drug alternatives available during this time period (1964–1967). Between 1920s and end of 1950s mainly barbiturates were used as anxiolytic drugs [22], but from the early 1960s these drugs were gradually replaced by the first-generation benzodiazepines such as chlordiazepoxide [23]. Hence, it is most likely that the mothers studied were prescribed either one of these two drug classes.

Since the thalidomide disaster in 1961, that stopped the prescription of this drug to pregnant women, numerous studies have investigated the effects of sedatives and other drugs as being teratogenic to the developing fetus. Anti-epileptic drugs have also shown teratogenic effects such as congenital malformations, i.e. phenobarbital at therapeutic dosages [24, 25]. Similarly, a study of migraine patients prescribed butalbital also reported an elevated risk of malformations [26].

According to a systematic review, chlordiazepoxide (and other benzodiazepines) have not been associated with any increased malformation risk [27]. This was supported by Wikner et al. [28] who, in addition, reported an increased risk of low birth weight and preterm birth following maternal use of benzodiazepines during pregnancy. Fetal breathing movements, necessary for lung development, have also previously been shown to be negatively affected by sedatives such as diazepam [29].

Our results indicate that the second trimester is the most sensitive time period for adverse influences of maternal sedative use in relation to offspring ARD. This is also the most critical period for lung development when cell proliferation and differentiation occur most rapidly [29, 30]. Hypothetically, the effect of sedating drugs upon ARD risk may partly be regarded as an adaptive response through their influence on lung development, fetal breathing and fetal growth during a sensitive time window of plasticity. As previously mentioned, other studies have shown association between growth retardation, low birth weight, and preterm birth on the one hand with risk of asthma and COPD on the other hand [6–9, 12]. However, when we adjusted for gestational age and birthweight, maternal use of sedative drugs still appeared as independently associated with ARD, while the SGA phenotype in itself was not (Supplemental Table S1).

Our finding that ARD is more common among female than male offspring is a confirmation of earlier studies [9, 31, 32]. Leynaert et al. [31] reported that asthma was 20% more frequent among women than men over 35 years, and that female sex is an independent risk marker for both non-allergic and allergic asthma.

Analysis stratified by sex showed an increased ARD risk among female offspring born LGA. Even though most studies have reported a positive association between low birth weight and increased risk of respiratory disease, our finding is a confirmation of Sin et al. [33] who demonstrated that children born large (> 4500 g) run a higher risk of childhood asthma. Consistent with our finding, obesity has been shown to be a risk marker for adult asthma among women exclusively, thus reflecting that sex differences may exist in ARD susceptibility according to body weight [34]. Indeed, increasing evidence suggests that the association between birth weight and risk of adult morbidity and mortality could reflect a U-shaped rather than a linear pattern [35].

In our study, maternal cigarette smoking during most of pregnancy predicted ARD. This is supported by previous studies [36–39]. The causal relationship between maternal smoking and offspring respiratory disease is complex since maternal smoking in pregnancy is also associated with mediating phenotypes such as IUGR/SGA [40, 41], preterm birth [40–43], low birth weight [40–43], and shorter birth height [43], which all in turn have been shown to affect (increase) risk of respiratory disease. Balte et al. [44] recently demonstrated that maternal gestational smoking showed no direct association with lung function in children and adolescents in a linear mixed model, but that in a linear path analysis direct negative associations were found with measures of lung function (Forced expiratory volume, FEV_1_/Forced vital capacity, FVC ratio, and Forced expiratory flow, FEF_25–75%_) at the age of 18 years. Additionally, maternal smoking showed an indirect association, through reduction in birthweight, with offspring FEV_1_ [44].

It is also possible that maternal smoking and use of sedatives both identify a group of mothers with lower socioeconomic status, psychosocial problems, proneness to mental illness, or physical stress during their pregnancy. Thus, maternal smoking and use of sedatives could at least partly be regarded as possible surrogate markers for adverse psychosocial conditions or low socioeconomic status. These factors could also indicate underlying averse maternal health or obstetric complications, specifically during the second trimester. Unfortunately, no information on these background variables were available for analysis. No synergistic effects on the risk of offspring ARD were observed for women who both smoked and used sedatives at the same time (data not shown). Since we lack information for these potential underlying factors our finding of sedative use as risk factor for ARD should be interpreted with caution.

## Limitations and strengths

There exist some obvious limitations when performing studies on historical cohorts like ours. The available variables were not collected for this study purpose and hence lack details about exact categories of sedative drugs used and dosages, as well as a risk of misclassification due to inexact documentation and reported data. Further, the only estimation possible of gestational length was from the women’s self-reported date of last menstrual period. The cohort also lacks information on important variables such as socioeconomic status and mental health of the mothers which could partly explain, or at least influence, the usage of sedative drugs and maternal smoking during pregnancy. Unfortunately we also lack information about emigration which could have affected the proportion of the cohort still available for study. We do not believe, however, that there is a large proportion of the offspring that has migrated from Sweden during the follow-up period.

*Second*, we used multiple categories of obstructive respiratory diseases that were considered and analysed together. Since asthma, chronic bronchitis, emphysema and bronchiectasis display different pathogeneses, and only share the clinical aspects of pulmonary obstructiveness, it could eventually be better to separate them for analysis in order to examine potential risk factors. However, we chose not to do so because the majority of affected offspring suffered from asthma, leaving too few individuals within the other diagnostic categories.

*Third*, offspring were considered affected by respiratory disease if they had either a diagnosis from the national registers, or were prescribed specific drugs used for treatment of respiratory diseases. This ensured that few individuals suffering from such conditions were missed although prescription of, for example, adrenergic broncho-dilating inhalers does not necessarily by default equalizes the diagnosis of a chronic respiratory disease since it can also be prescribed as symptomatic relief treatment in conditions such as acute respiratory infections or allergic reactions. Even the diagnoses chosen by clinicians, obtained from the national registers, may risk misclassification bias. However, the prevalence of respiratory disease in our study (20.4%) matches well the combined estimated mean prevalence of asthma (10%) and COPD (9%) in Sweden [45].

On the other hand, there are also numerous strengths of our study. The cohort offers objective measurements of birth weight, birth length, placental weight, gestational age, pre-eclampsia, and mode of delivery together with comprehensive self-reported data on maternal smoking, infection episodes, as well as sedative drug and antibiotic use. Approximately 50% of the mothers smoked during pregnancy and/or used sedative drugs, an unusual and very high proportion reflecting historical and cultural circumstances in the early 1960ies in Southern Sweden. The follow-up time is more than 50 years, and there are few historical cohorts to match this with detailed exposure data.

Associations of congenital malformations and pharmaceutic use during pregnancy are presently monitored, while the latter’s associations with adult disease are not. The explanation is that national registers of drug usage during pregnancy currently has a limited follow-up time. Thus such research is dependent on historical cohorts like the HbgBC. However, in the future adult disease should be included in risk analyses of pharmaceuticals influencing fetal development and outcomes.

## Conclusions

In summary, we report a significant and independent association between maternal use of sedative drugs during pregnancy (i.e. 2nd trimester) with higher risk of offspring ARD. Maternal smoking throughout most of pregnancy, and offspring female sex, were also factors associated with ARD. When stratified for sex, maternal use of sedative drugs during pregnancy remained the only significant risk marker shared by both sexes. Further studies are required to confirm the negative effect of sedative use in pregnancy upon ARD risk in the offspring.

## Electronic supplementary material

Below is the link to the electronic supplementary material.

Supplementary material 1 (DOCX 151 kb)

## Data Availability

The authors confirm that the data supporting the findings in this study are available within the article and its supplementary materials. By present ethical approval, data is restricted to this specific research group and only within Sweden. Analyses in IBM SPSS Statistics were performed without syntaxes, outputs are available by request.

## References

[1] Xie M, Liu X, Cao X, Guo M, Li X (2020). Trends in prevalence and incidence of chronic respiratory diseases from 1990 to 2017. Respir Res.

[2] López-Campos JL, Tan W, Soriano JB (2016). Global burden of COPD. Respirology.

[3] Barker DJ, Osmond C (1986). Infant mortality, childhood nutrition, and ischaemic heart disease in England and Wales. Lancet.

[4] Barker DJ, Winter PD, Osmond C, Margetts B, Simmonds SJ (1989). Weight in infancy and death from ischaemic heart disease. Lancet.

[5] Law CM, Barker DJP (1994). Fetal influences on blood pressure. J Hypertens.

[6] Barker DJP, Godfrey KM, Fall C, Barker DJP (1992). Relation of birthweight and childhood respiratory infection to adult lung function and death from chronic airways disease. Fetal and infant origins of adult disease.

[7] Barker DJP, Osmond C, Law CM, Barker DJP (1992). Intrauterine and early postnatal origins of cardiovascular disease and chronic bronchitis. Fetal and infant origins of adult disease.

[8] Barker DJP, Barker DJP (1992). Intrauterine origins of cardiovascular and obstructive lung disease in adult life. Fetal and infant origins of adult disease.

[9] Broström EB, Akre O, Katz-Salamon M, Jaraj D, Kaijser M (2013). Obstructive pulmonary disease in old age among individuals born preterm. Eur J Epidemiol.

[10] Lawlor DA, Ebrahim S, Davey Smith V (2005). Association of birth weight with adult lung function: findings from the British Women’s Heart and Health Study and a meta-analysis. Thorax.

[11] Hancox RJ, Poulton R, Greene JM, McLachlan CR, Pearce MS, Sears MR (2009). Associations between birth weight, early childhood weight gain and adult lung function. Thorax.

[12] Canoy D, Pekkanen J, Elliott P, Pouta A, Laitinen J, Hartikainen AL (2007). Early growth and adult respiratory function in men and women followed from the fetal period to adulthood. Thorax.

[13] Mu M, Ye S, Bai MJ, Liu GL, Tong Y, Wang SF (2014). Birth weight and subsequent risk of asthma: a systematic review and meta-analysis. Heart Lung Circ.

[14] Briana DD, Malamitsi-Puchner A (2013). Small for gestational age birth weight: impact on lung structure and function. Paediatr Respir Rev.

[15] Hanson MA, Gluckman PD (2014). Early developmental conditioning of later health and disease: physiology or pathophysiology?. Physiol Rev.

[16] Nilsson PM, Hofvendahl S, Hofvendahl E, Brandt L, Ekbom A (2006). Smoking in pregnancy in relation to gender and adult mortality risk in offspring: the Helsingborg Birth Cohort Study. Scand J Public Health.

[17] Palmgren B, Wahlén T, Wallander B (1973). Toxemia and cigarette smoking during pregnancy. Prospecitve consecutive investigation of 3927 pregnancies. Acta Obstet Gynecol Scand.

[18] Marsal K, Persson PH, Larsen T, Lilja H, Selbing A, Sultan B (1996). Intrauterine growth curves based on ultrasonically estimated foetal weights. Acta Paediatr.

[19] Chung WS, Lai CY, Lin CL, Kao CH (2015). Adverse respiratory events associated with hypnotics use in patients of chronic obstructive pulmonary disease: a population-based case-control study. Medicine (Baltimore).

[20] Nakafero G, Sanders RD, Nguyen-Van-Tam JS, Myles PR (2015). Association between benzodiazepine use and exacerbations and mortality in patients with asthma: a matched case-control and survival analysis using the United Kingdom Clinical Practice Research Datalink. Pharmacoepidemiol Drug Saf.

[21] Baillargeon J, Singh G, Kuo YG, Raji MA, Westra J, Sharma G (2019). Association of opiod and benzodiazepine use with adverse respiratory events in older adults with chronic obstructive pulmonary disease. Ann Am Thorac Soc.

[22] López-Munoz F, Ucha-Udabe R, Álamo C (2005). The history of barbiturates a century after their clinical introduction. Neuropsychiatr Dis Treat.

[23] López-Muñoz F, Alamo C, García-García P (2011). The discovery of chlordiazepoxide and the clinical introduction of benzodiazepines: half a century of anxiolytic drugs. J Anxiety Disord.

[24] Tomson T, Battino D, Bonizzoni E, Lindhout D, Sabers A, Perucca E, Vajda F, EURAP Study Group (2011). Dose-dependent risk of malformations with antiepileptic drugs: an analysis of data from the EURAP epilepsy and pregnancy registry. Lancet Neurol.

[25] Kaneko S, Battino D, Andermann E, Wada K, Kan R, Takeda A, Nakane Y (1999). Congenital malformations due to antiepileptic drugs. Epilepsy Res.

[26] Browne ML, Van Zutphen AR, Botto LD, Louik C, Richardson S, Druschel CM (2014). Maternal butalbital use and selected defects in the national birth defects prevention study. Headache.

[27] Iqbal MM, Sobhan T, Ryals T (2002). Effects of commonly used benzodiazepines on the fetus, the neonate, and the nursing infant. Psychiatr Serv.

[28] Wikner BN, Stiller C-O, Bergman U, Asker C, Källén B (2007). Use of benzodiazepines and benzodiazepine receptor agonists during pregnancy: neonatal outcome and congenital malformations. Pharmacoepidemiol Drug Saf.

[29] Kotecha S (2000). Lung growth for beginners. Paediatr Respir Rev.

[30] Wigglesworth JS (1988). Lung development in the second trimester. Br Med Bull.

[31] Leynaert B, Sunyer J, Garcia-Esteban R, Svanes C, Jarvis D, Cerveri I (2012). Gender differences in prevalence, diagnosis and incidence of allergic and non-allergic asthma: a population-based cohort. Thorax.

[32] Aryal S, Diaz-Guzman E, Mannino DM (2013). COPD and gender differences: an up-date. Transl Res.

[33] Sin DD, Spier S, Svenson LW, Schopflocher DP, Senthilselvan A, Cowie RL (2004). The relationship between birth weight and childhood asthma: a population-based cohort study. Arch Pediatr Adolesc Med.

[34] Hancox RJ, Milne BJ, Poulton R, Taylor DR, Greene JM, McLachlan CR (2005). Sex differences in the relation between body mass index and asthma and atopy in a birth cohort. Am J Respir Crit Care Med.

[35] Baker JL, Olsen LW, Sørensen TIA (2008). Weight at birth and all-cause mortality in adulthood. Epidemiology.

[36] Landau LI (2008). Tobacco smoke exposure and tracking of lung function into adult life. Paediatr Respir Rev.

[37] Beyer D, Mitfessel H, Gillissen A (2009). Maternal smoking promotes chronic obstructive lung disease in the offspring as adults. Eur J Med Res.

[38] Hayatbakhsh MR, Sadasivam S, Mamun AA, Najman JM, Williams GM, O’Callaghan MJ (2009). Maternal smoking during and after pregnancy and lung function in early adulthood: a prospective study. Thorax.

[39] Savran O, Ulrik CS (2018). Early life insults as determinants of chronic obstructive pulmonary disease in adult life. Int J Chronic Obstr Pulm Dis.

[40] Horta BL, Victora CG, Menezes AM, Halpern R, Barros FC (1997). Low birthweight, preterm births and intrauterine growth retardation in relation to maternal smoking. Paediatr Perinat Epidemiol.

[41] Ko TJ, Tsai LY, Chu LC, Yeh SJ, Leung C, Chen CY (2014). Parental smoking during pregnancy and its association with low birth weight, small for gestational age, and preterm birth offspring: a birth cohort study. Pediatr Neonatol.

[42] Leonardi-Bee J, Smyth A, Britton J, Coleman T (2008). Environmental tobacco smoke and fetal health: systematic review and meta-analysis. Arch Dis Child Fetal Neonatal Ed.

[43] Crane JM, Keough M, Murphy P, Burrage L, Hutchens D (2011). Effects of environmental tobacco smoke on perinatal outcomes: a retrospective cohort study. BJOG.

[44] Balte P, Karmaus W, Roberts G, Kurukulaaratchy R, Mitchell F, Arshad H (2016). Relationship between birth weight, maternal smoking during pregnancy and childhood and adolescent lung function: a path analysis. Respir Med.

[45] Borna E, Nwaru BI, Bjerg A, Mincheva R, Rådinger M, Lundbäck B, Ekerljung L (2019). Changes in the prevalence of asthma and respiratory symptoms in western Sweden between 2008 and 2016. Allergy.

