# Supplementary material for: Early life exposures and risk of adult respiratory disease during 50 years of follow-up
Source: Eur J Epidemiol. 2020 Apr 8;35(12):1157–66. doi: 10.1007/s10654-020-00626-3 (PMC7762746; doi:10.1007/s10654-020-00626-3)
Supplement: Supplementary file 1 — Supplementary material 1 (DOCX 151 kb) [file 10654_2020_626_MOESM1_ESM.docx]

**SUPPLEMENTAL TABLES and FIGURES**

| **Supplemental Table S1.** The association of sedative use and adult respiratory disease (diagnosis or medication) in relation to trimester of use. | | | | | | | | | |  |
| --- | --- | --- | --- | --- | --- | --- | --- | --- | --- | --- |
|  |  |  | |  |  |  |  |  |  |  |
|  | OR* (95% CI) | *P* | |  | aOR† (95% CI) | *P* |  | aOR‡ (95% CI) | *P* |  |
| **1) Maternal use of sedative drugs (*n*=3675)** |  |  | |  |  |  |  |  |  |  |
| No sedatives used during pregnancy (*n*=1887) | *reference* | | |  | *reference* | |  | *reference* | |  |
| Sedatives used during pregnancy in any trimester (1788) | 1.13 (0.96–1.33) | | 0.13 |  | 1.21 (1.02-1.43) | **0.03** |  | 1.20 (1.02-1.43) | **0.03** |  |
|  |  | |  |  |  |  |  |  |  |  |
| **2) Maternal use of sedative drugs (*n*=2215)** |  | |  |  |  |  |  |  |  |  |
| No sedatives used during pregnancy (n=1887) | *reference* | | |  | *reference* | |  | *reference* | |  |
| During 2:nd trimester with or without use in other trimesters (*n*=328) | 1.32 (1.0–1.74) | | 0.051 |  | 1.41 (1.06-1.88) | **0.02** |  | 1.41 (1.05-1.88) | **0.02** |  |
| (1460 excluded) |  | |  |  |  |  |  |  |  |  |
|  |  | |  |  |  |  |  |  |  |  |
| **3) Maternal use of sedative drugs (*n*=1945)** |  | |  |  |  |  |  |  |  |  |
| No sedatives used during pregnancy (*n*=1887) | *reference* | | |  | *reference* | |  | *reference* |  |  |
| During 2:nd trimester only (*n*=58) | 2.36 (1.36–4.08) | | **0.002** |  | 2.65 (1.50-4.69) | **0.001** |  | 2.69 (1.51-4.79) | **0.001** |  |
| (1730 excluded) |  | |  |  |  |  |  |  |  |  |
|  |  | |  |  |  |  |  |  |  |  |
| **4) Maternal use of sedative drugs (*n*=3405)** |  | |  |  |  |  |  |  |  |  |
| No sedatives used during pregnancy (*n*=1887) | *Reference* | | |  | *reference* | |  | *reference* | |  |
| During 2:nd trimester only (*n*=58) | 2.36 (1.36–4.08 | | **0.002** |  | 2.75 (1.56-4.85) | **<0.001** |  | 2.71 (1.52-4.82 | **0.001** |  |
| During 1:st and/or 3:rd trimester (*n*=1460) | 1.09 (0.92–1.30) | | 0.31 |  | 1.16 (0.97-1.39) | 0.10 |  | 1.16 (0.97-1.38) | 0.12 |  |
| (270 excluded) |  | |  |  |  |  |  |  |  |  |
|  |  | |  |  |  |  |  |  |  |  |
| **5) Maternal use of sedative drugs (*n*=3675)** |  | |  |  |  |  |  |  |  |  |
| No sedatives used during pregnancy (*n*=1887) | *Reference* | | |  | *reference* | |  | *reference* | |  |
| During 2:nd trimester with or without use in other trimesters (*n*=328) | 1.32 (0.1.0–1.74) | | 0.051 |  | 1.45 (1.09-1.94) | **0.01** |  | 1.45 (1.08-1.93) | **0.01** |  |
| During 1:st and/or 3:rd trimester (*n*=1460) | 1.09 (0.92–1.30) | | 0.31 |  | 1.16 (0.97-1.38) | 0.11 |  | 1.15 (0.96-1.38) | 0.13 |  |
|  |  | |  |  |  |  |  |  |  |  |
| **6) Maternal use of sedative drugs (*n*=3675)** |  | |  |  |  |  |  |  |  |  |
| No sedatives used during pregnancy (*n*=1887) | *Reference* | | |  | *reference* | |  | *reference* | |  |
| During 2:nd trimester only (*n*=58) | 2.36 (1.36–4.08) | | **0.002** |  | 2.76 (1.57-4.86) | **<0.001** |  | 2.70 (1.52-4.79) | **<0.001** |  |
| During 1:st and/or 3:rd trimester (*n*=1460) | 1.09 (0.92–1.30) | | 0.31 |  | 1.16 (0.97-1.38) | 0.11 |  | 1.15 (0.96-1.38) | 0.13 |  |
| During 2:nd and any other trimester (*n*=270) | 1.14 (0.83–1.55) | | 0.42 |  | 1.23 (0.89-1.70) | 0.20 |  | 1.24 (0.90-1.71) | 0.19 |  |
| OR, odds ratio; 95% CI, 95 % confidence interval; aOR, adjusted odds ratio | | | | | | | | | | |
| *Univariate logistic regression | | | | | | | | | | |
| † ***Model 1:*** Adjusted for offspring sex, birth height, birth weight, gestational age of delivery, weight group, maternal sedative usage, maternal smoking, maternal preeclampsia, and maternal infections | | | | | | | | | |  |
| ‡ ***Model 2:*** Adjusted for offspring sex, weight group, prematurity, born in winter, maternal sedative usage, maternal smoking, maternal preeclampsia, maternal infections, and mode of delivery | | | | | | | | | | |

**Supplemental Table S2.** Univariate and adjusted associations between early life risk factors and offspring diagnosed with asthma.

|  | **HR* (95% CI)** | ***P*** |  | **aHR† (95% CI)** | ***P*** |
| --- | --- | --- | --- | --- | --- |
| **Sex** |  |  |  |  |  |
| Female | 1.3 (1.0–1.9) | 0.09 |  | 1.3 (0.9–1.9) | 0.09 |
| Male | *reference* |  |  | *reference* |  |
| **Birth height (cm)** | 1.0 (0.99–1.0) | 0.4 |  | 1.0 (0.99–1.0) | 0.5 |
| **Maternal use of sedatives** |  |  |  |  |  |
| No sedatives used during pregnancy | *reference* |  |  | *reference* |  |
| During 1^st^ trimester | 0.7 (0.2–2.2) | 0.5 |  | 0.7 (0.2–2.2) | 0.5 |
| During 2^nd^ trimester | 3.0 (1.3–6.9) | **0.01** |  | 3.0 (1.3–6.8) | **0.01** |
| During 3^rd^ trimester | 1.0 (0.7–1.5) | 0.9 |  | 1.0 (0.7–1.5) | 0.9 |
| During all trimesters | 1.4 (0.6–3.0) | 0.4 |  | 1.4 (0.7–3.1) | 0.4 |
| **Maternal Infections** |  |  |  |  |  |
| No infections during pregnancy | *reference* |  |  | *reference* |  |
| Any infection during pregnancy | 1.1 (0.7–1.6) | 0.7 |  | 1.1 (0.7–1.7) | 0.6 |
| **Maternal Smoking** |  |  |  |  |  |
| No | *reference* |  |  | *reference* |  |
| Smoking during 1^st^ trimester | 0.5 (0.2–1.6) | 0.3 |  | 0.5 (0.2–1.7) | 0.3 |
| Smoking during most of pregnancy | 1.0 (0.7–1.4) | 0.8 |  | 0.99 (0.7–1.4) | 0.95 |
| **Birth weight categories** |  |  |  |  |  |
| Small for gestational age (SGA) | 0.7 (0.4–1.3) | 0.2 |  | 0.6 (0.3–1.3) | 0.2 |
| Appropriate for gestational age (AGA) | *reference* |  |  | *reference* |  |
| Large for gestational age (LGA) | 0.7 (0.4–14) | 0.4 |  | 0.7 (0.4–1.4) | 0.3 |
| HR, Hazard ratio; 95% CI, 95 % confidence interval; aHR, adjusted Hazard ratio | | | | | |
| * Univariate analysis | | | | | |
| † Adjusted for sex, maternal use of sedatives and maternal smoking (common cause approach) | | | | | |

| **Supplemental Table S3.** Univariate and adjusted associations between early life risk factors and offspring diagnosed with other lung disease (*n*=41). | | | | | |  |
| --- | --- | --- | --- | --- | --- | --- |
|  | HR* (95% CI) | *P* |  | aHR† (95% CI) | *P* | |
| **Sex** |  |  |  |  |  | |
| Female | 1.4 (0.7–2.5 | 0.3 |  | 1.6 (0.8–3.1) | 0.2 | |
| Male | *reference* |  |  | *reference* |  | |
| **Birth height (cm)** | 1.0 (0.99–1.0) | 0.2 |  | 1.0 (0.99–1.0) | 0.5 | |
| **Maternal use of sedatives** |  |  |  |  |  | |
| No sedatives used during pregnancy | *reference* |  |  | *reference* |  | |
| During 1^st^ trimester | 4.1 (1.4–12.2) | **0.01** |  | 4.1 (1.4–12.2) | 0.1 | |
| During 2^nd^ trimester | . | . |  | . | . | |
| During 3^rd^ trimester | 1.3 (0.6–2.7) | 0.5 |  | 1.3 (0.7–2.7) | 0.4 | |
| During all trimesters | 2.6 (0.8–9.0) | 0.1 |  | 2.7 (0.8–9.3) | 0.1 | |
| **Maternal Infections** |  |  |  |  |  | |
| No infections during pregnancy | *reference* |  |  | *reference* |  | |
| Any infection during pregnancy | 1.3 (0.7–2.8) | 0.4 |  | 1.1 (0.5–2.4) | 0.8 | |
| **Maternal Smoking** |  |  |  |  |  | |
| No | *reference* |  |  | *reference* |  | |
| Smoking during 1^st^ trimester | 1.2 (0.3–5.3) | 0.8 |  | 1.4 (0.3–6.0) | 0.7 | |
| Smoking during most of pregnancy | 1.1 (0.6–2.1) | 0.7 |  | 1.2 (0.6–2.3) | 0.6 | |
| **Birth weight categories** |  |  |  |  |  | |
| Small for gestational age (SGA) | 0.7 (0.2–2.4) | 0.6 |  | 0.8 (0.2–2.6) | 0.7 | |
| Appropriate for gestational age (AGA) | *reference* |  |  | *reference* |  | |
| Large for gestational age (LGA) | 1.2 (0.5–3.1) | 0.7 |  | 1.2 (0.4–3.5) | 0.7 | |
| HR, Hazard ratio; 95% CI, 95 % confidence interval; aHR, adjusted Hazard ratio | | | | | | |
| * Univariate analysis | | | | | | |
| † Adjusted for sex, maternal use of sedatives and maternal smoking (common cause approach) | | | | | | |

| **Supplemental Table S4.** Univariate and adjusted associations between early life risk factors and offspring prescribed medication for treatment of ARD (*n*=718). | | | | | |
| --- | --- | --- | --- | --- | --- |
|  |  |  |  |  |  |
|  |  |  |  |  |  |
|  | **HR* (95% CI)** | ***P*** |  | **aHR† (95% CI)** | ***P*** |
| **Sex** |  |  |  |  |  |
| Female | 1.5 (1.3–1.8) | **<0.001** |  | 1.5 (1.3–1.8) | **<0.001** |
| Male | *reference* |  |  | *reference* |  |
| **Birth height (cm)** | 1.0 (0.99–1.0) | **0.01** |  | 1.0 (1.0–1.0) | 0.2 |
| **Maternal use of sedatives** |  |  |  |  |  |
| No sedatives used during pregnancy | *reference* |  |  | *reference* |  |
| During 1^st^ trimester | 0.8 (0.5–1.3) | 0.3 |  | 0.8 (0.5–1.3) | 0.3 |
| During 2^nd^ trimester | 2.2 (1.4–3.4) | **0.001** |  | 2.2 (1.4–3.4) | **0.001** |
| During 3^rd^ trimester | 1.1 (0.9–1.3) | 0.2 |  | 1.1 (1.0–1.3) | 0.2 |
| During all trimesters | 1.4 (1.0–1.9) | 0.09 |  | 1.4 (1.0–2.0) | **0.05** |
| **Maternal Infections** |  |  |  |  |  |
| No infections during pregnancy | *reference* |  |  | *reference* |  |
| Any infection during pregnancy | 0.9 (0.8–1.1) | 0.5 |  | 0.9 (0.8–1.1) | 0.4 |
| **Maternal Smoking** |  |  |  |  |  |
| No | *reference* |  |  | *reference* |  |
| Smoking during 1^st^ trimester | 0.9 (0.6–1.4) | 0.6 |  | 0.9 (0.6–1.3) | 0.5 |
| Smoking during most of pregnancy | 1.2 (1.0–1.4) | **0.02** |  | 1.2 (1.0–1.4) | **0.02** |
| **Birth weight categories** |  |  |  |  |  |
| Small for gestational age (SGA) | 1.2 (0.9–1.5) | 0.2 |  | 1.1 (0.9–1.4) | 0.4 |
| Appropriate for gestational age (AGA) | *reference* |  |  | *reference* |  |
| Large for gestational age (LGA) | 1.1 (0.9–1.4) | 0.3 |  | 1.2 (0.9–1.5) | 0.2 |
| HR, Hazard ratio; 95% CI, 95 % confidence interval; aHR, adjusted Hazard ratio | | | | |  |
| * Univariate analysis |  |  |  |  |  |
| † Adjusted for sex, maternal use of sedatives and maternal smoking (common cause approach). | | | | |  |


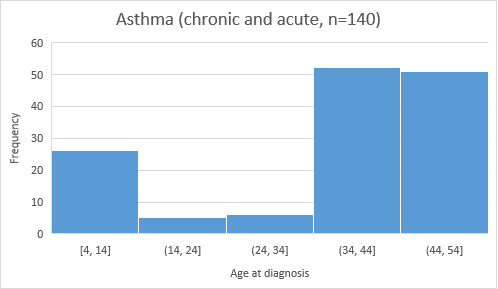


**Supplemental Figure S1.** Age distribution of first recorded diagnosis of asthma.


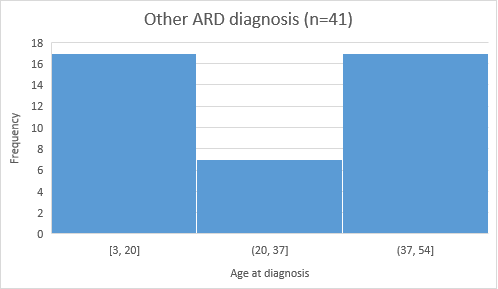


**Supplemental Figure S2.** Age distribution of first recorded diagnosis of other ARD.


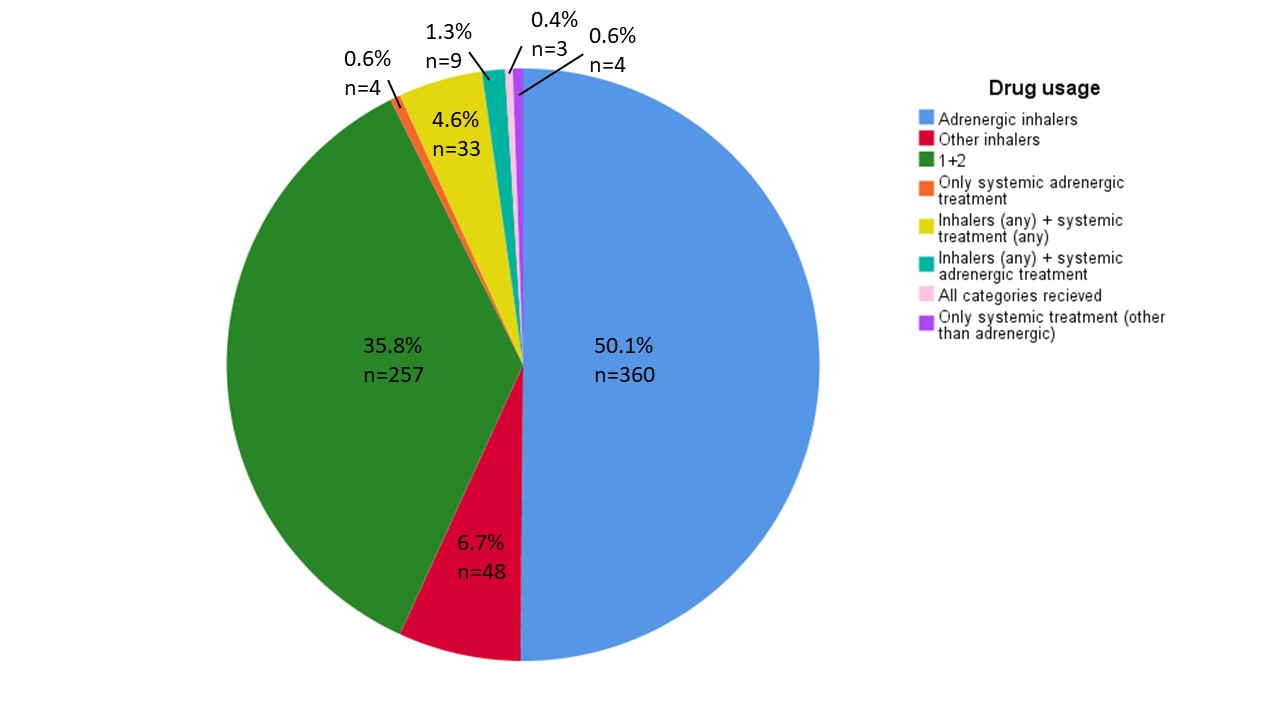


**Supplemental Figure S3.** Distribution of drugs used for respiratory disorders and related conditions (n and percentages).


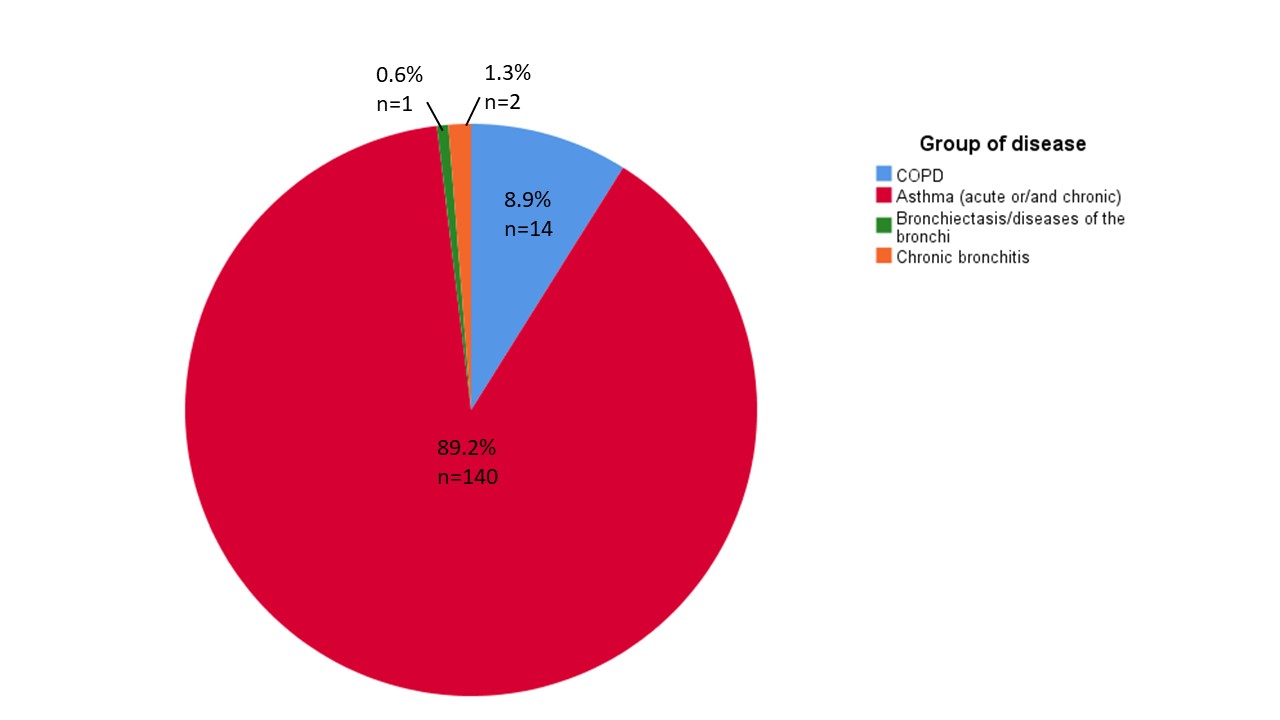


**Supplemental Figure S4.** Distribution of respiratory diseases according to ICD-8, ICD-9, and ICD-10 classification of diagnoses (n and percentages).
